# 4-(4-Bromo­phen­yl)-2,3,3a,4,5,11c-hexa­hydro­benzo[*f*]furo[3,2-*c*]quinoline

**DOI:** 10.1107/S1600536811031084

**Published:** 2011-08-11

**Authors:** Nan Wu, Rongli Zhang, Xinnian Li, Xin Xu, Zhou Xu

**Affiliations:** a Department of Aviation Oil and Materials, Xuzhou Airforce College, Xuzhou Jiangsu 221110, People’s Republic of China; b Department of Chemistry, Xuzhou Medical College, Xuzhou Jiangsu 221004, People’s Republic of China

## Abstract

In the title compound, C_21_H_18_BrNO, both heterocyclic rings, *viz.* the hydro­pyridine ring and the adjacent hydro­furan ring, adopt envelope conformations. These two heterocycles make a dihedral angle of 37.3 (1)°. The dihedral angle between the hydro­pyridine and benzene rings is 69.6 (1)°. In the crystal, adjacent mol­ecules are linked by pairs of inter­molecular C—H⋯O hydrogen bonds, forming centrosymmetric dimers.

## Related literature

For the biological properties of quinoline derivatives, see: Nesterova *et al.* (1995 ▶); Yamada *et al.* (1992 ▶); Faber *et al.* (1984 ▶); Johnson *et al.* (1989 ▶). For related structures, see: Ramesh *et al.* (2008 ▶); Zhao & Teng (2008 ▶); Bai *et al.* (2009 ▶); Du *et al.* (2010 ▶); Wang *et al.* (2010 ▶).
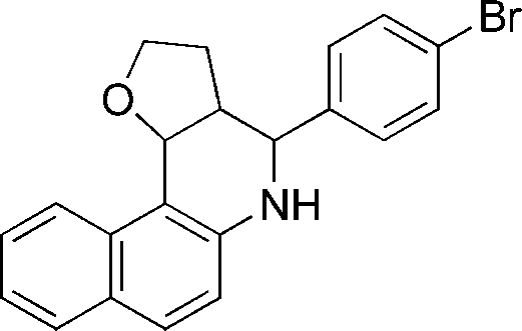


## Experimental

### 

#### Crystal data

C_21_H_18_BrNO
*M* *_r_* = 380.27
Triclinic, 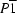

*a* = 9.4019 (2) Å
*b* = 9.6025 (2) Å
*c* = 10.4660 (2) Å
α = 103.888 (1)°
β = 114.075 (1)°
γ = 92.469 (1)°
*V* = 826.81 (3) Å^3^
*Z* = 2
Mo *K*α radiation
μ = 2.49 mm^−1^
*T* = 296 K
0.20 × 0.09 × 0.04 mm

#### Data collection

Bruker APEXII area-detector diffractometer
Absorption correction: multi-scan (*SADABS*; Bruker, 2001 ▶) *T*_min_ = 0.793, *T*_max_ = 0.899
10901 measured reflections
2921 independent reflections
2301 reflections with *I* > 2σ(*I*)
*R*_int_ = 0.037

#### Refinement

*R*[*F* ^2^ > 2σ(*F* ^2^)] = 0.033
*wR*(*F* ^2^) = 0.076
*S* = 1.04
2921 reflections
221 parameters
H atoms treated by a mixture of independent and constrained refinement
Δρ_max_ = 0.22 e Å^−3^
Δρ_min_ = −0.32 e Å^−3^

### 

Data collection: *SMART* (Bruker, 2001 ▶); cell refinement: *SAINT* (Bruker, 2001 ▶); data reduction: *SAINT*; program(s) used to solve structure: *SHELXS97* (Sheldrick, 2008 ▶); program(s) used to refine structure: *SHELXL97* (Sheldrick, 2008 ▶); molecular graphics: *SHELXTL* (Sheldrick, 2008 ▶); software used to prepare material for publication: *SHELXTL*.

## Supplementary Material

Crystal structure: contains datablock(s) global, I. DOI: 10.1107/S1600536811031084/zb2015sup1.cif

Structure factors: contains datablock(s) I. DOI: 10.1107/S1600536811031084/zb2015Isup2.hkl

Supplementary material file. DOI: 10.1107/S1600536811031084/zb2015Isup3.cml

Additional supplementary materials:  crystallographic information; 3D view; checkCIF report

## Figures and Tables

**Table 1:** Hydrogen-bond geometry (Å, °)

*D*—H⋯*A*	*D*—H	H⋯*A*	*D*⋯*A*	*D*—H⋯*A*
C9—H9⋯O1^i^	0.93	2.69	3.462 (3)	141

Symmetry code: (i) 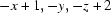.

**Fig. 1.**
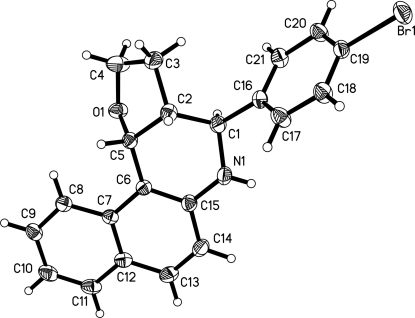
The molecular structure drawing shows 30% probability of displacement ellipsoids and the atom-numbering scheme.

**Fig. 2.**
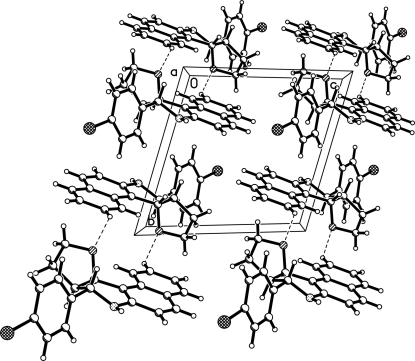
The molecular packing diagram of (I). Dashed lines indicate hydrogen bonds of type C9—H9···O1 which links the adjacent molecules forming dimmers along a.

## supplementary crystallographic information

### Comment

Quinoline derivatives has been extensively studied due to its varies of biological properties, such as psychotropic activity (Nesterova, *et al.*, 1995), anti-allergic (Yamada *et al.*, 1992) and anti-inflammatory activity (Faber *et al.*, 1984 and Johnson *et al.*, 1989). The title compound (Fig. 1), may be used as a new precursor for obtaining bioactive molecules. Herein, we report the crystal structure of the title compound, (I).

In the crystal structure of (I), the hydropyridine ring of the furoquinoline moiety adopts an envelope conformation (Fig. 1). The atom C1 deviates from the plane defined by the atoms C2/C5/C6/C15/N1 by 0.646 (3) Å. This conformation is different from those reported in other hydropyridine derivatives (For related structrues, see Ramesh, *et al.*, 2008; Zhao & Teng, 2008; Bai *et al.*, 2009; Du, *et al.*, 2010). In the adjacent hydrofuran ring, the atoms C2—C4 and O1 are coplanar, while the atom C5 deviates from the plane by 0.522 (3) Å. This data indicates that the above hydrofuran ring also adopts an envelope conformation. These two heterocycles make a dihedral angle of 37.3 (1)°. The basal plane of the hydropyridine ring is nearly coplanar to the naphthalene ring C6—C15, forming a dihedral angle of 6.0 (1)°. The dihedral angle between the phenyl and the hydropyridine ring is 69.6 (1)°. The hydrogen bond of C9—H9···O1 links the adjacent moleclues forming dimmers along *a* axis (Figure 2). This hydrogen bonding pattern is same with the one reported in literature (Wang *et al.*, 2010).

### Experimental

The title compound, (I), was prepared by the reaction of 4-bromobenzaldehyde (0.361 g, 2.0 mmol), naphthalen-2-amine (0.286 g, 2.0 mmol), 2,3-dihydrofuran (0.252 g, 3.0 mmol), I_2_ (0.026 g, 0.1 mmol) and THF (10 ml) for 18 h (yield 87%, mp. 523–525 K). Crystals of (I) suitable for X-ray diffraction were obtained by slow evaporation of a DMF solution.

### Refinement

The H atoms were calculated geometrically and refined as riding, with C—H = 0.93–0.98 Å, except for H1, and with *U*_iso_(H) = 1.2*U*_eq_(parent atom).

### Crystal data

**Table:**

C_21_H_18_BrNO	*Z* = 2
*M**_r_* = 380.27	*F*(000) = 388
Triclinic, *P*1	*D*_x_ = 1.527 Mg m^−^^3^
Hall symbol: -P 1	Melting point = 523–525 K
*a* = 9.4019 (2) Å	Mo *K*α radiation, λ = 0.71073 Å
*b* = 9.6025 (2) Å	Cell parameters from 2885 reflections
*c* = 10.4660 (2) Å	θ = 2.2–22.9°
α = 103.888 (1)°	µ = 2.49 mm^−^^1^
β = 114.075 (1)°	*T* = 296 K
γ = 92.469 (1)°	Block, colourless
*V* = 826.81 (3) Å^3^	0.20 × 0.09 × 0.04 mm

### Data collection

**Table:**

Bruker APEXII area-detector diffractometer	2921 independent reflections
Radiation source: fine-focus sealed tube	2301 reflections with *I* > 2σ(*I*)
graphite	*R*_int_ = 0.037
φ and ω scans	θ_max_ = 25.0°, θ_min_ = 2.2°
Absorption correction: multi-scan (*SADABS*; Bruker, 2001)	*h* = −11→11
*T*_min_ = 0.793, *T*_max_ = 0.899	*k* = −11→11
10901 measured reflections	*l* = −12→12

### Refinement

**Table:**

Refinement on *F*^2^	Primary atom site location: structure-invariant direct methods
Least-squares matrix: full	Secondary atom site location: difference Fourier map
*R*[*F*^2^ > 2σ(*F*^2^)] = 0.033	Hydrogen site location: inferred from neighbouring sites
*wR*(*F*^2^) = 0.076	H atoms treated by a mixture of independent and constrained refinement
*S* = 1.04	*w* = 1/[σ^2^(*F*_o_^2^) + (0.0327*P*)^2^ + 0.242*P*] where *P* = (*F*_o_^2^ + 2*F*_c_^2^)/3
2921 reflections	(Δ/σ)_max_ = 0.001
221 parameters	Δρ_max_ = 0.22 e Å^−^^3^
0 restraints	Δρ_min_ = −0.32 e Å^−^^3^

### Special details

**Table:**

Geometry. All e.s.d.'s (except the e.s.d. in the dihedral angle between two l.s. planes) are estimated using the full covariance matrix. The cell e.s.d.'s are taken into account individually in the estimation of e.s.d.'s in distances, angles and torsion angles; correlations between e.s.d.'s in cell parameters are only used when they are defined by crystal symmetry. An approximate (isotropic) treatment of cell e.s.d.'s is used for estimating e.s.d.'s involving l.s. planes.
Refinement. Refinement of *F*^2^ against ALL reflections. The weighted *R*-factor *wR* and goodness of fit *S* are based on *F*^2^, conventional *R*-factors *R* are based on *F*, with *F* set to zero for negative *F*^2^. The threshold expression of *F*^2^ > σ(*F*^2^) is used only for calculating *R*-factors(gt) *etc*. and is not relevant to the choice of reflections for refinement. *R*-factors based on *F*^2^ are statistically about twice as large as those based on *F*, and *R*- factors based on ALL data will be even larger.

### Fractional atomic coordinates and isotropic or equivalent isotropic displacement parameters (Å^2^)

**Table:**

	*x*	*y*	*z*	*U*_iso_*/*U*_eq_	
Br1	1.51042 (4)	0.37481 (4)	0.64000 (4)	0.05334 (14)	
O1	0.7657 (2)	0.0345 (2)	0.8561 (2)	0.0463 (5)	
C16	1.1935 (3)	0.2498 (3)	0.8548 (3)	0.0360 (6)	
C19	1.3806 (3)	0.3222 (3)	0.7264 (3)	0.0364 (6)	
N1	1.1406 (3)	0.3070 (3)	1.0684 (3)	0.0457 (7)	
C6	0.8940 (3)	0.2371 (3)	1.0725 (3)	0.0335 (6)	
C1	1.0888 (3)	0.2098 (3)	0.9222 (3)	0.0387 (7)	
H1B	1.0959	0.1099	0.9283	0.046*	
C21	1.2412 (3)	0.1435 (3)	0.7724 (3)	0.0430 (7)	
H21	1.2100	0.0462	0.7605	0.052*	
C12	0.8804 (3)	0.2858 (3)	1.3089 (3)	0.0387 (7)	
C20	1.3343 (3)	0.1788 (3)	0.7073 (3)	0.0446 (7)	
H20	1.3647	0.1063	0.6515	0.054*	
C15	1.0518 (3)	0.2965 (3)	1.1442 (3)	0.0377 (7)	
C7	0.8052 (3)	0.2325 (3)	1.1546 (3)	0.0330 (6)	
C5	0.8148 (3)	0.1891 (3)	0.9095 (3)	0.0350 (6)	
H5	0.7211	0.2361	0.8765	0.042*	
C2	0.9162 (3)	0.2214 (3)	0.8341 (3)	0.0400 (7)	
H2	0.9075	0.3181	0.8190	0.048*	
C17	1.2421 (3)	0.3929 (3)	0.8702 (3)	0.0480 (8)	
H17	1.2106	0.4661	0.9242	0.058*	
C8	0.6415 (3)	0.1764 (3)	1.0879 (3)	0.0383 (7)	
H8	0.5893	0.1401	0.9869	0.046*	
C9	0.5593 (3)	0.1746 (3)	1.1694 (3)	0.0434 (7)	
H9	0.4519	0.1385	1.1231	0.052*	
C11	0.7913 (4)	0.2808 (3)	1.3891 (3)	0.0478 (8)	
H11	0.8407	0.3153	1.4903	0.057*	
C10	0.6344 (4)	0.2263 (3)	1.3208 (3)	0.0491 (8)	
H10	0.5776	0.2237	1.3753	0.059*	
C14	1.1242 (3)	0.3524 (3)	1.2979 (3)	0.0473 (8)	
H14	1.2300	0.3943	1.3452	0.057*	
C18	1.3365 (3)	0.4302 (3)	0.8073 (3)	0.0453 (7)	
H18	1.3695	0.5273	0.8199	0.054*	
C13	1.0424 (4)	0.3462 (3)	1.3777 (3)	0.0471 (8)	
H13	1.0932	0.3820	1.4788	0.057*	
C3	0.8387 (4)	0.1035 (4)	0.6873 (3)	0.0576 (9)	
H3A	0.7779	0.1450	0.6097	0.069*	
H3B	0.9180	0.0569	0.6639	0.069*	
C4	0.7334 (4)	−0.0034 (4)	0.7062 (3)	0.0610 (9)	
H4A	0.6236	0.0002	0.6472	0.073*	
H4B	0.7538	−0.1011	0.6758	0.073*	
H1A	1.234 (4)	0.323 (3)	1.114 (3)	0.052 (10)*	

### Atomic displacement parameters (Å^2^)

**Table:**

	*U*^11^	*U*^22^	*U*^33^	*U*^12^	*U*^13^	*U*^23^
Br1	0.0485 (2)	0.0682 (2)	0.0570 (2)	0.00705 (16)	0.03258 (16)	0.02427 (18)
O1	0.0560 (12)	0.0410 (12)	0.0405 (12)	−0.0063 (10)	0.0247 (10)	0.0045 (10)
N1	0.0306 (14)	0.0645 (18)	0.0388 (15)	−0.0024 (13)	0.0144 (12)	0.0120 (14)
C1	0.0387 (16)	0.0397 (16)	0.0401 (17)	0.0031 (13)	0.0190 (14)	0.0126 (14)
C2	0.0389 (16)	0.0452 (17)	0.0399 (17)	0.0026 (13)	0.0179 (13)	0.0182 (15)
C3	0.0471 (18)	0.083 (3)	0.0358 (18)	−0.0093 (17)	0.0177 (15)	0.0092 (18)
C4	0.082 (2)	0.053 (2)	0.046 (2)	−0.0024 (18)	0.0342 (18)	0.0023 (17)
C5	0.0334 (15)	0.0381 (16)	0.0351 (16)	0.0025 (12)	0.0156 (12)	0.0122 (14)
C6	0.0351 (15)	0.0335 (15)	0.0318 (15)	0.0037 (12)	0.0141 (12)	0.0099 (13)
C7	0.0399 (15)	0.0264 (14)	0.0353 (15)	0.0054 (12)	0.0186 (13)	0.0089 (13)
C8	0.0417 (16)	0.0342 (16)	0.0405 (17)	0.0027 (13)	0.0202 (13)	0.0093 (14)
C9	0.0457 (17)	0.0352 (16)	0.057 (2)	0.0028 (13)	0.0314 (16)	0.0097 (15)
C10	0.066 (2)	0.0423 (18)	0.055 (2)	0.0022 (16)	0.0432 (18)	0.0111 (16)
C11	0.068 (2)	0.0406 (18)	0.0398 (18)	0.0021 (16)	0.0305 (17)	0.0085 (15)
C12	0.0494 (17)	0.0340 (16)	0.0348 (16)	0.0040 (13)	0.0200 (14)	0.0105 (14)
C13	0.0525 (19)	0.0485 (19)	0.0331 (16)	0.0013 (15)	0.0138 (15)	0.0084 (15)
C14	0.0383 (17)	0.0534 (19)	0.0400 (18)	−0.0042 (14)	0.0112 (14)	0.0074 (16)
C15	0.0348 (15)	0.0414 (17)	0.0380 (16)	0.0038 (13)	0.0162 (13)	0.0126 (14)
C16	0.0328 (15)	0.0365 (16)	0.0410 (17)	0.0043 (12)	0.0169 (13)	0.0134 (14)
C17	0.059 (2)	0.0356 (17)	0.063 (2)	0.0094 (15)	0.0400 (17)	0.0116 (16)
C18	0.0505 (18)	0.0342 (16)	0.059 (2)	0.0015 (14)	0.0324 (16)	0.0116 (16)
C19	0.0318 (14)	0.0437 (17)	0.0367 (16)	0.0046 (13)	0.0162 (13)	0.0141 (14)
C20	0.0472 (17)	0.0413 (18)	0.0517 (19)	0.0104 (14)	0.0297 (15)	0.0087 (15)
C21	0.0442 (17)	0.0337 (16)	0.0562 (19)	0.0061 (13)	0.0255 (15)	0.0146 (15)

### Geometric parameters (Å, °)

**Table:**

Br1—C19	1.905 (3)	C7—C8	1.420 (4)
O1—C4	1.420 (3)	C5—C2	1.527 (3)
O1—C5	1.436 (3)	C5—H5	0.9800
C16—C17	1.379 (4)	C2—C3	1.534 (4)
C16—C21	1.386 (4)	C2—H2	0.9800
C16—C1	1.510 (3)	C17—C18	1.383 (4)
C19—C18	1.366 (4)	C17—H17	0.9300
C19—C20	1.368 (4)	C8—C9	1.367 (4)
N1—C15	1.381 (3)	C8—H8	0.9300
N1—C1	1.454 (4)	C9—C10	1.391 (4)
N1—H1A	0.80 (3)	C9—H9	0.9300
C6—C15	1.379 (4)	C11—C10	1.362 (4)
C6—C7	1.428 (3)	C11—H11	0.9300
C6—C5	1.494 (4)	C10—H10	0.9300
C1—C2	1.529 (4)	C14—C13	1.355 (4)
C1—H1B	0.9800	C14—H14	0.9300
C21—C20	1.384 (4)	C18—H18	0.9300
C21—H21	0.9300	C13—H13	0.9300
C12—C11	1.414 (4)	C3—C4	1.497 (4)
C12—C13	1.415 (4)	C3—H3A	0.9700
C12—C7	1.417 (4)	C3—H3B	0.9700
C20—H20	0.9300	C4—H4A	0.9700
C15—C14	1.415 (4)	C4—H4B	0.9700
			
C4—O1—C5	105.9 (2)	C5—C2—C3	102.0 (2)
C17—C16—C21	117.7 (2)	C1—C2—C3	112.5 (2)
C17—C16—C1	121.3 (2)	C5—C2—H2	110.3
C21—C16—C1	121.0 (2)	C1—C2—H2	110.3
C18—C19—C20	121.5 (2)	C3—C2—H2	110.3
C18—C19—Br1	118.6 (2)	C16—C17—C18	121.6 (3)
C20—C19—Br1	119.9 (2)	C16—C17—H17	119.2
C15—N1—C1	118.9 (2)	C18—C17—H17	119.2
C15—N1—H1A	117 (2)	C9—C8—C7	121.3 (3)
C1—N1—H1A	113 (2)	C9—C8—H8	119.4
C15—C6—C7	119.5 (2)	C7—C8—H8	119.4
C15—C6—C5	119.7 (2)	C8—C9—C10	120.7 (3)
C7—C6—C5	120.6 (2)	C8—C9—H9	119.6
N1—C1—C16	109.9 (2)	C10—C9—H9	119.6
N1—C1—C2	107.6 (2)	C10—C11—C12	121.2 (3)
C16—C1—C2	112.3 (2)	C10—C11—H11	119.4
N1—C1—H1B	109.0	C12—C11—H11	119.4
C16—C1—H1B	109.0	C11—C10—C9	119.9 (3)
C2—C1—H1B	109.0	C11—C10—H10	120.1
C20—C21—C16	121.5 (3)	C9—C10—H10	120.1
C20—C21—H21	119.3	C13—C14—C15	121.3 (3)
C16—C21—H21	119.3	C13—C14—H14	119.3
C11—C12—C13	122.0 (3)	C15—C14—H14	119.3
C11—C12—C7	119.4 (3)	C19—C18—C17	118.9 (3)
C13—C12—C7	118.6 (3)	C19—C18—H18	120.5
C19—C20—C21	118.8 (3)	C17—C18—H18	120.5
C19—C20—H20	120.6	C14—C13—C12	120.7 (3)
C21—C20—H20	120.6	C14—C13—H13	119.6
C6—C15—N1	121.3 (3)	C12—C13—H13	119.6
C6—C15—C14	119.9 (3)	C4—C3—C2	105.5 (2)
N1—C15—C14	118.8 (2)	C4—C3—H3A	110.6
C12—C7—C8	117.5 (2)	C2—C3—H3A	110.6
C12—C7—C6	119.9 (2)	C4—C3—H3B	110.6
C8—C7—C6	122.6 (2)	C2—C3—H3B	110.6
O1—C5—C6	110.8 (2)	H3A—C3—H3B	108.8
O1—C5—C2	104.9 (2)	O1—C4—C3	107.6 (3)
C6—C5—C2	115.6 (2)	O1—C4—H4A	110.2
O1—C5—H5	108.4	C3—C4—H4A	110.2
C6—C5—H5	108.4	O1—C4—H4B	110.2
C2—C5—H5	108.4	C3—C4—H4B	110.2
C5—C2—C1	111.1 (2)	H4A—C4—H4B	108.5
			
C15—N1—C1—C16	174.5 (2)	C7—C6—C5—C2	170.7 (2)
C15—N1—C1—C2	52.0 (3)	O1—C5—C2—C1	−88.2 (3)
C17—C16—C1—N1	−41.8 (3)	C6—C5—C2—C1	34.2 (3)
C21—C16—C1—N1	139.6 (3)	O1—C5—C2—C3	31.8 (3)
C17—C16—C1—C2	77.9 (3)	C6—C5—C2—C3	154.2 (2)
C21—C16—C1—C2	−100.6 (3)	N1—C1—C2—C5	−55.6 (3)
C17—C16—C21—C20	−0.1 (4)	C16—C1—C2—C5	−176.6 (2)
C1—C16—C21—C20	178.5 (3)	N1—C1—C2—C3	−169.2 (2)
C18—C19—C20—C21	−0.3 (4)	C16—C1—C2—C3	69.7 (3)
Br1—C19—C20—C21	179.4 (2)	C21—C16—C17—C18	−0.6 (4)
C16—C21—C20—C19	0.6 (4)	C1—C16—C17—C18	−179.2 (3)
C7—C6—C15—N1	−178.0 (2)	C12—C7—C8—C9	0.4 (4)
C5—C6—C15—N1	−2.8 (4)	C6—C7—C8—C9	−179.4 (2)
C7—C6—C15—C14	−0.1 (4)	C7—C8—C9—C10	−0.9 (4)
C5—C6—C15—C14	175.1 (2)	C13—C12—C11—C10	178.0 (3)
C1—N1—C15—C6	−23.0 (4)	C7—C12—C11—C10	−0.3 (4)
C1—N1—C15—C14	159.1 (3)	C12—C11—C10—C9	−0.1 (4)
C11—C12—C7—C8	0.2 (4)	C8—C9—C10—C11	0.7 (4)
C13—C12—C7—C8	−178.2 (2)	C6—C15—C14—C13	1.5 (4)
C11—C12—C7—C6	−180.0 (2)	N1—C15—C14—C13	179.4 (3)
C13—C12—C7—C6	1.6 (4)	C20—C19—C18—C17	−0.4 (4)
C15—C6—C7—C12	−1.4 (4)	Br1—C19—C18—C17	179.9 (2)
C5—C6—C7—C12	−176.5 (2)	C16—C17—C18—C19	0.9 (4)
C15—C6—C7—C8	178.4 (2)	C15—C14—C13—C12	−1.3 (4)
C5—C6—C7—C8	3.3 (4)	C11—C12—C13—C14	−178.6 (3)
C4—O1—C5—C6	−164.3 (2)	C7—C12—C13—C14	−0.3 (4)
C4—O1—C5—C2	−38.9 (3)	C5—C2—C3—C4	−13.9 (3)
C15—C6—C5—O1	114.7 (3)	C1—C2—C3—C4	105.2 (3)
C7—C6—C5—O1	−70.2 (3)	C5—O1—C4—C3	29.8 (3)
C15—C6—C5—C2	−4.4 (4)	C2—C3—C4—O1	−8.8 (4)

### Hydrogen-bond geometry (Å, °)

**Table:**

*D*—H···*A*	*D*—H	H···*A*	*D*···*A*	*D*—H···*A*
C9—H9···O1^i^	0.93	2.69	3.462 (3)	141.

Symmetry codes: (i) −*x*+1, −*y*, −*z*+2.
